# 嵌段共聚温敏亲和色谱固定相的制备及其对抗体的分离纯化

**DOI:** 10.3724/SP.J.1123.2023.09028

**Published:** 2023-12-08

**Authors:** Dongmei GUO, Yiran XIA, ur RAHMAN Mujeeb, Jianzhong WANG, Jiawei LIU, Quan BAI

**Affiliations:** 合成与天然功能分子教育部重点实验室,西北大学现代分离科学研究所, 陕西省现代分离科学重点实验室,西北大学化学与材料科学学院,陕西西安710127; Key Laboratory of Synthetic and Natural Functional Molecule Chemistry of Ministry of Education, Institute of Modern Separation Science, Key Laboratory of Modern Separation Science in Shaanxi Province, College of Chemistry and Materials Science, Northwest University, Xi’an 710127, China

**Keywords:** 温敏色谱, 亲和色谱, 抗体分离, 聚(*N*-异丙基丙烯酰胺), 4-乙烯基吡啶, temperature-responsive chromatography, affinity chromatography, antibody separation, poly(*N*-isopropylacrylamide), 4-vinylpyridine

## Abstract

抗体药物在癌症治疗和免疫诊断中起着重要作用,但抗体的分离纯化通常采用酸性洗脱,易导致抗体聚集失活等问题。本研究以硅胶为基质,以温敏嵌段聚合物聚[(*N*-异丙基丙烯酰胺)-*b*-4-乙烯基吡啶](P[NIPAM-*b*-4VP])为间隔臂,以4-巯基乙基吡啶(MEP)为配基,制备了一种嵌段共聚温敏亲和色谱固定相SiO_2_-P[NIPAM-*b*-4VP]-MEP,并以牛血清白蛋白(BSA)和*γ*-球蛋白为模型蛋白,对制备的温敏亲和色谱固定相的色谱性能进行了表征。分别考察了流动相盐浓度和温度对二者分离性能的影响。结果表明,在40 ℃时该固定相只选择性保留*γ*-球蛋白,而不保留BSA;在5 ℃时采用含0.6 mol/L NaCl的Tris-HCl(pH 8.0)缓冲溶液进行洗脱,*γ*-球蛋白的质量回收率为92.7%。该固定相对*γ*-球蛋白的吸附量为(71.5±2.1) mg/g(*n*=3),是传统温敏亲和色谱固定相SiO_2_-PNIPAM-MEP的2倍。将该固定相用于人血清中IgG的分离纯化,仅通过改变流动相温度和盐浓度即可一步实现对抗体的分离纯化,纯度大于97.4%±0.7%。上述结果表明该温敏亲和色谱固定相不仅对抗体具有特异的选择性,而且洗脱条件温和,绿色环保,从根本上解决了传统亲和色谱采用酸性洗脱易使抗体蛋白变性失活的难题。该技术在抗体药物的分离纯化和工业生产中具有重要的应用价值。

## Abstract (en)

Antibodies play an essential role in cancer diagnosis and treatment because of the specificity for target biomolecules and reduction of side effects. However, antibodies separation and purification still face some challenges. Antibody elution from columns using a low-pH aqueous solution leads to aggregation or loss of activity of the antibody drugs. In this paper, a block copolymer-based temperature-responsive affinity chromatography (TRAC) stationary phase, SiO_2_-P[NIPAM-*b*-4VP]-MEP using the block temperature-responsive copolymer poly(*N*-isopropylacrylamide-*b*-4-vinylpyridine) (P[NIPAM-*b*-4VP]) as the space arms and 4-mercaptoethyl pyridine (MEP) as the ligand was prepared for antibody separation. The TRAC column was tested using bovine serum albumin (BSA) and *γ*-globulin as model proteins, and the effects of salt concentration in the mobile phase and temperature on their separation were studied in detail. At 40 ℃, the TRAC stationary phase only selectively retained *γ*-globulin due to the specific affinity interaction between antibodies and the ligand MEP. At 5 ℃, *γ*-globulin can be eluted from the column with a mass recovery of 92.7% using a Tris-HCl buffer (pH 8.0) solution containing 0.6 mol/L NaCl. The adsorption capacity of *γ*-globulin on this stationary phase was (71.5 ±2.1) mg/g (*n*=3), which was twice that of a traditional temperature-sensitive affinity chromatography stationary phase SiO_2_-PNIPAM-MEP. The stationary phase was also used to separate and purify immunoglobulin (IgG) in human serum in one step by altering the temperature and ion strength of the mobile phase, resulting in a purity of 97.4%±0.7%. Thus, this new technology has specific selectivity for antibodies, as well as mild and green elution conditions, ultimately resolving the problem of traditional affinity chromatography using acid elution, which can lead to the antibodies aggregation/inactivation. This technology has great application potential for the industrial production of antibody drugs.

抗体药物因其靶向性强、特异性高、毒副作用小、疗效好,已广泛用于癌症等疑难杂症的治疗。由于抗体原料液的复杂性及对抗体产品高纯度的要求,抗体的分离纯化成为抗体药物生产的关键,约占抗体生产成本的50%~80%^[1]^。目前抗体的分离纯化主要采用蛋白A亲和层析^[2⇓⇓-5]^或其他色谱分离技术,如亲硫色谱^[6]^、疏水电荷诱导色谱^[7,8]^、混合模式色谱^[9]^及仿生多肽亲和色谱^[10]^等,但抗体蛋白通常需在酸性条件下进行洗脱,易导致抗体聚集失活。这些聚集体必须除去,这不仅增加操作单元和生产成本,还降低了产量。

温敏色谱是近年来发展起来的新型色谱分离技术^[11]^。它是将温敏聚合物,如聚(*N*-异丙基丙烯酰胺)(PNIPAM)修饰到固定相表面,利用PNIPAM对环境温度的响应特性,以纯水作为流动相,通过调节柱温来改变固定相表面的亲疏水性,实现对目标物的有效分离^[12]^。温敏色谱分离条件温和,避免使用有机溶剂,绿色环保,其最低临界溶液温度(LCST)为32 ℃,且其相变行为不易受其他环境因素(如pH或盐浓度)的影响,在接近生理条件下分离不会破坏生物大分子的活性,为生物大分子的分离提供了新思路^[13,14]^。

Ooi等^[15]^将PNIPAM与配体4-巯基乙基吡啶(MEP)偶联后接枝到琼脂糖微球,制备了一种温敏亲和吸附剂用于抗体分离。该吸附剂在40 ℃可吸附*γ*-球蛋白,在5 ℃可解吸90%的*γ*-球蛋白(*γ*-globulin)。证明了利用温敏亲和吸附剂通过调控温度可对抗体蛋白进行有效吸附和解吸。随后,该课题组^[16]^将PNIPAM与4-乙烯基吡啶(4-VP)共聚,将温敏嵌段共聚物P[NIPAM-*b*-4VP]接枝到聚(甲基丙烯酸缩水甘油酯)(PGMA)聚合物微球上,制备了一种温度和pH双控响应色谱分离介质用于抗体分离,但*γ*-球蛋白必须在5 ℃酸性(pH 3.0)条件下才能完全洗脱。Nagase等^[17]^用PNIPAM和聚(4-乙烯基吡啶)(P4VP)对二氧化硅表面进行改性,制备了一种混合聚合物刷改性的温敏色谱固定相。利用混合聚合物刷的特殊性质,只需改变柱温即可分离利妥昔单抗和牛血清白蛋白(BSA)。此外,该课题组^[18]^还制备了温敏阴离子交换聚合物刷修饰的温敏离子交换色谱固定相,通过温度调节抗体蛋白与固定相之间的疏水和静电相互作用力,实现了对西妥昔单抗、贝伐单抗和利妥昔单抗混合物的分离。最近,我们课题组^[19]^以MEP为配体,以硅胶为基质,以温敏树枝状聚合物聚酰胺-胺-PNIPAM(PAMAM-PNIPAM)为间隔臂,制备了高容量的温敏仿生亲和色谱固定相,构建了一种用于抗体分离纯化的高容量、绿色环保的温敏仿生亲和色谱方法。该方法以纯水为流动相,BSA和*γ*-球蛋白在40 ℃上样,5 ℃进行洗脱,仅通过改变温度可一步实现对二者的完全分离。将其应用于人血清中IgG和蛋黄中IgY的分离纯化,一步纯化后IgG和IgY的纯度为99%,质量回收率大于90%。但该固定相对BSA和*γ*-球蛋白均有保留,对抗体蛋白的特异选择性较低。

研究表明,将PNIPAM与亲水或疏水性单体共聚成嵌段共聚物可调节温敏聚合物的亲疏水性^[20]^。P4VP是一种两亲性聚合物,具有悬垂的可电离吡啶基团,这些吡啶环提供了与蛋白质的多个相互作用位点,作为固定相配基用于抗体的分离纯化^[16,21]^。为了进一步提高温敏亲和色谱固定相对抗体的选择性,本研究将4-VP与PNIPAM共聚,以硅胶为基质,制备嵌段共聚温敏亲和固定相SiO_2_-P[NIPAM-*b*-4VP]-MEP。以BSA和*γ*-球蛋白为模型蛋白,对制备的温敏亲和固定相的色谱性能进行了表征。分别考察了流动相盐浓度和温度对二者分离性能的影响。结果表明,该温敏亲和色谱固定相在40 ℃时可选择性保留*γ*-球蛋白,而不保留BSA,仅通过改变温度和盐浓度在5 ℃时即可一步实现对抗体的分离纯化。该温敏亲和色谱固定相不仅对抗体表现出特异的选择性,而且洗脱条件温和,绿色环保,从根本上解决了传统亲和色谱采用酸性洗脱易使抗体蛋白变性失活的难题。

## 1 实验部分

### 1.1 仪器、试剂与材料

高效液相色谱仪(LC10A, Shimadzu,日本), X-射线光电子能谱仪(PH15000 Versa Probe Ⅲ, Ulvac,日本),傅里叶变换红外光谱仪(TENSOR, Bruker,德国),凝胶渗透色谱仪(GPC, Agilent1260 Infinity Ⅱ,安捷伦,美国)。

硅胶微球(5 μm,孔径30 nm)购于中国科学院兰州化学物理研究所。*N*-异丙基丙烯酰胺、4-VP、三(2-羧乙基)膦盐酸盐(TCEP)、安息香双甲醚(DMPA)购于萨恩化学技术(上海)有限公司。偶氮二异丁腈(AIBN)购于上海山浦化工有限公司。1-乙基-(3-二甲基氨基丙基)碳酰二亚胺盐酸盐(EDC·HCl)和*N*-羟基琥珀酰亚胺(NHS)购于上海思域化工科技有限公司。*N*,*N*-二甲基甲酰胺(DMF)购于成都科隆化学品有限公司。BSA和*γ*-球蛋白(来源于牛血)购于Sigma-Aldrich(美国)。

### 1.2 温敏嵌段共聚物P[NIPAM-*b*-4VP]的制备

依据文献[15],采用可逆加成-断裂转移法(RAFT)制备3 kDa的温敏聚合物-链转移剂(PNIPAM-CTA)。将3.15 g PNIPAM-CTA和8.0 mL DMF加入25 mL圆底烧瓶中搅拌溶解,再分别加入14.53 g AIBN和1.05 g 4-VP,接入双排管,充分排气后将圆底烧瓶移入70 ℃油浴中反应18 h。取出烧瓶置于冰水浴中猝灭反应,用截留1 kDa的透析袋对反应产物透析3 d,然后冷冻干燥除去产物中的水分,得到温敏嵌段共聚物P[NIPAM-*b*-4VP]-CTA^[16]^。

### 1.3 嵌段温敏亲和配基P[NIPAM-*b*-4VP]-MEP的制备

将0.86 g 的P[NIPAM-*b*-4VP]-CTA和4 mL DMF加入到25 mL圆底烧瓶中,搅拌溶解后再加入6.2 mg的TCEP,通入N_2_ 5 min后,再加入0.15 g的正己胺,继续通入N_2_搅拌反应3 h,将P[NIPAM-*b*-4VP]-CTA中的三硫酯完全还原成巯基,溶液颜色变为深绿色。用锡纸包裹烧瓶,在避光条件下加入6 mg光催化剂DMPA和0.15 g 4-VP,通入N_2_ 15 min,在365 nm的紫外光照射下搅拌3 h。反应结束后,溶液变为棕色。用截留1 kDa的透析袋将产物透析3 d,冷冻干燥后得到P[NIPAM-*b*-4VP]-MEP。

### 1.4 嵌段温敏亲和色谱固定相SiO_2_-P[NIPAM-*b*-4VP]-MEP的制备

将0.35 g P[NIPAM-*b*-4VP]-MEP和40 mL去离子水加入到100 mL三颈瓶中,冰水浴中搅拌使其完全溶解。再加入0.59 g的EDC·HCl和0.36 g的NHS,继续搅拌2 h后加入1.2 g的氨基化硅胶SiO_2_-NH_2_, 25 ℃搅拌反应24 h。反应结束后,以10000 r/min转速离心10 min,依次用蒸馏水和甲醇洗涤产物3次,在30 ℃真空干燥箱中干燥12 h,得到嵌段温敏亲和色谱固定相SiO_2_-P[NIPAM-*b*-4VP]-MEP。

### 1.5 色谱柱装填及色谱条件

将0.8 g制备的温敏亲和色谱固定相SiO_2_-P[NIPAM-*b*-4VP]-MEP超声分散到25 mL甲醇中,采用匀浆法在35 MPa压力下装填入50 mm×4.6 mm的不锈钢色谱柱内。

将色谱柱浸入40 ℃恒温水浴内恒温,采用流动相A液(50 mmol/L Tris-HCl, pH 8.0)平衡色谱柱30 min,进样10 μL, 10 min后停泵。将色谱柱移至5 ℃恒温水浴中恒温10 min,然后用在5 ℃预冷的流动相B液(50 mmol/L Tris-HCl+0.6 mol/L NaCl, pH 8.0)等度洗脱。流速为1.0 mL/min,检测波长280 nm。

### 1.6 吸附等温线的测定

分别准确称取10份20 mg温敏亲和色谱固定相材料于10个2 mL离心管中,随后依次向其中加入1 mL不同质量浓度(0.1~1.0 mg/mL)的*γ*-球蛋白溶液。将离心管置于不同温度(10、20、30和40 ℃)的恒温摇床中以1000 r/min恒温振荡1 h。吸附平衡后,以10000 r/min转速离心10 min,收集上清液。采用Bradford法测定各上清液中*γ*-球蛋白的含量,依据下式计算温敏亲和色谱固定相对*γ*-球蛋白的吸附量:

*Q*_e_=(*C*_0_-*C*_e_)*V/m*

式中,*C*_0_为初始蛋白含量(mg/mL), *C*_e_为平衡时蛋白含量(mg/mL), *V*为蛋白溶液总体积(mL), *m*为所用吸附剂质量(g)。

### 1.7 SDS-PAGE电泳分析

收集色谱馏分,冷冻干燥后,用100 μL去离子水溶解。取15.6 μL样品溶液于2 mL离心管中,再加入4.4 μL样品溶解液(不含二硫苏糖醇),将离心管置于沸水中加热3 min,冷却后离心,取上清液上样10 μL。采用10%分离胶和8%浓缩胶进行非还原SDS-PAGE电泳分析,浓缩胶分离电压为90 V,分离胶分离电压为180 V。电泳结束后,采用考马斯亮蓝染色3~4 h,使用脱色液完全脱色后,利用凝胶成像系统分析*γ*-球蛋白的含量和纯度。

## 2 结果与讨论

### 2.1 嵌段温敏亲和色谱固定相SiO_2_-P[NIPAM-*b*-4VP]-MEP的表征

嵌段温敏亲和色谱固定相SiO_2_-P[NIPAM-*b*-4VP]-MEP的合成过程如图1所示。首先将线性温敏聚合物PNIPAM-CTA与4-VP共聚制备温敏嵌段共聚物P[NIPAM-*b*-4VP]-CTA,然后利用TCEP将三硫酯还原为巯基,再利用“巯-烯”点击化学与4-VP反应,在P[NIPAM-*b*-4VP]的末端构建亲和配基MEP,最后将嵌段温敏亲和配体P[NIPAM-*b*-4VP]-MEP与氨基硅胶反应,制备得到嵌段共聚温敏亲和色谱固定相SiO_2_-P[NIPAM-*b*-4VP]-MEP。

**图1:**
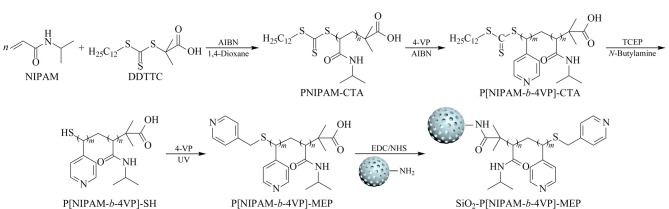
嵌段共聚温敏亲和色谱固定相SiO_2_-P[NIPAM-*b*-4VP]-MEP的制备流程图

利用GPC测定了温敏聚合物PNIPAM-CTA和温敏嵌段共聚物P[NIPAM-*b*-4VP]-CTA的分子质量,分别为3148 Da和9272 Da,后者较前者的分子质量增加约6000 Da,证明PNIPAM与4-VP成功实现共聚。结合^1^H-NMR测试结果,经计算PNIPAM的聚合度*m*为28, P4VP的聚合度*n*为56。

分别利用FT-IR和XPS对所制备的嵌段温敏亲和色谱固定相的结构进行了表征。图2为嵌段温敏亲和配基P[NIPAM-*b*-4VP]-MEP和温敏亲和固定相SiO_2_-P[NIPAM-*b*-4VP]-MEP的FT-IR图。从图中可看出,在P[NIPAM-*b*-4VP]-MEP中,1640 cm^-1^处为羰基的特征吸收峰,1550 cm^-1^处对应的峰是酰胺Ⅱ带的特征峰,1495 cm^-1^和1398 cm^-1^处是吡啶基团的特征振动峰,表明4-VP成功地与温敏聚合物PNIPAM共聚为嵌段聚合物。当温敏亲和配基通过羰基与硅胶表面的氨基反应后,除上述特征峰保留外,在472、807和1096 cm^-1^处还出现了Si-O-Si的弯曲和伸缩振动峰,表明嵌段温敏亲和配基已成功键合到硅胶表面。

**图2:**
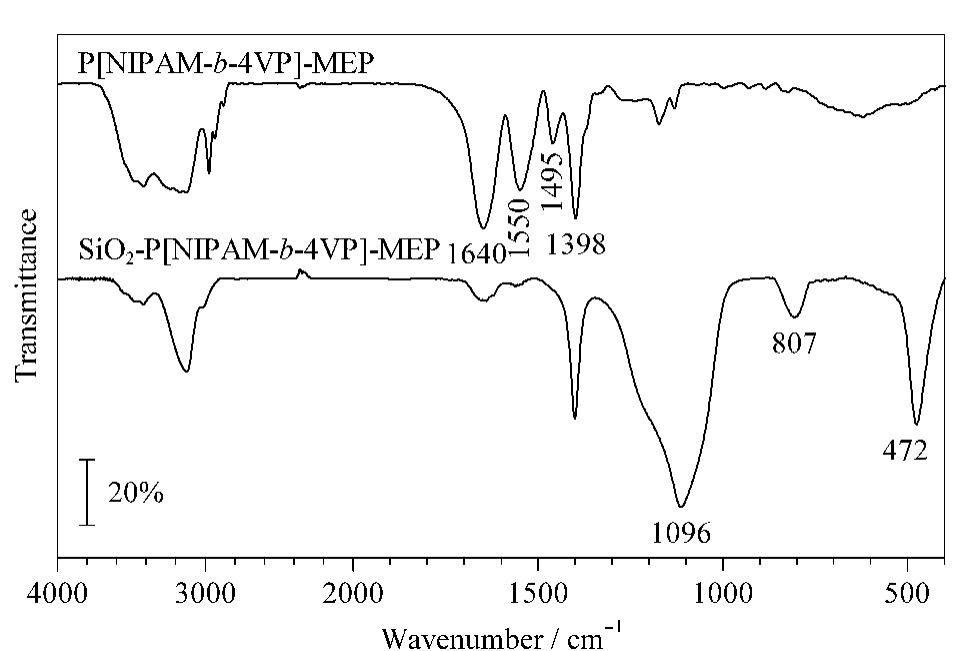
P[NIPAM-*b*-4VP]-MEP和SiO_2_-P[NIPAM-*b*-4VP]-MEP的FT-IR图

图3是SiO_2_、SiO_2_-NH_2_和SiO_2_-P[NIPAM-*b*-4VP]-MEP的XPS图。从图中可以看出,固定相SiO_2_-P[NIPAM-*b*-4VP]-MEP除含有与SiO_2_-NH_2_相同的特征峰外,如O 1*s*(532.2 eV)、N 1*s*(400.2 eV)、C 1*s*(283.4 eV)、Si 2*p*(102.3 eV)和Si 2*s*(153.2 eV)等,还含有S元素的特征峰S 2*s* (226.1 eV)和S 2*p* (164.3 eV)。表1中列出了利用XPS测得的SiO_2_-NH_2_、SiO_2_-PNIPAM-MEP与SiO_2_-P[NIPAM-*b*-4VP]-MEP中各元素的含量。从表1中可以看出,接枝了嵌段温敏共聚物的固定相SiO_2_-P[NIPAM-*b*-4VP]-MEP中C、O、S、N等元素的含量均高于接枝线性温敏聚合物的固定相SiO_2_-PNIPAM-MEP,进一步表明PNIPAM与4-VP共聚为嵌段共聚物,且成功接枝到硅胶表面。

**图3:**
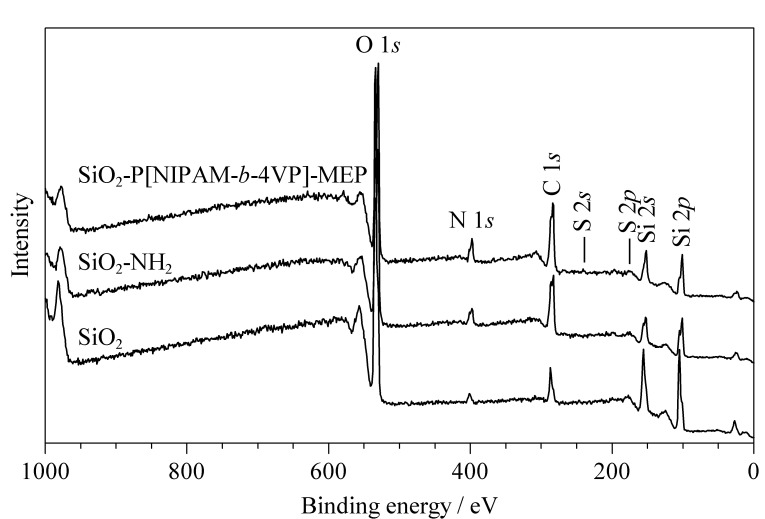
SiO_2_、SiO_2_-NH_2_和SiO_2_-P[NIPAM-*b*-4VP]-MEP的XPS图

**表1:** SiO_2_-NH_2_、SiO_2_-PNIPAM-MEP和SiO_2_-P[NIPAM- *b*-4VP]-MEP的元素分析

Sample	Atomic percents/%				
	C 1*s*	O 1*s*	Si 2*p*	S 2*p*	N 1*s*
SiO_2_-NH_2_	14.49	25.44	58.28	0	1.79
SiO_2_-PNIPAM-MEP	38.17	37.7	14.94	2.10	7.09
SiO_2_-P[NIPAM-*b*-4VP]-MEP	40.13	36.25	13.04	2.06	8.52

### 2.2 温敏亲和色谱固定相对BSA和*γ*-球蛋白的分离

由于PNIPAM的疏水性会因聚合物的水化和脱水随外界温度的变化而变化,因此接枝了温敏聚合物PNIPAM的温敏色谱固定相的表面性质很容易通过改变柱温而改变^[11]^。当温度高于其LCST时,固定相表面由于PNIPAM的脱水卷曲收缩而呈疏水性,相反,当温度低于其LCST时,PNIPAM链的伸展水化导致固定相表面呈亲水性,因此可通过改变色谱柱的温度来调节分析物与固定相之间的亲疏水作用,从而将其分离。我们分别以线性温敏聚合物PNIPAM和温敏嵌段共聚物P[NIPAM-*b*-4VP]为间隔臂,以MEP为配体,制备了两种温敏亲和色谱固定相SiO_2_-PNIPAM-MEP与SiO_2_-P[NIPAM-*b*-4VP]-MEP,将其用于BSA与*γ*-球蛋白的分离,结果如图4所示。

**图4:**
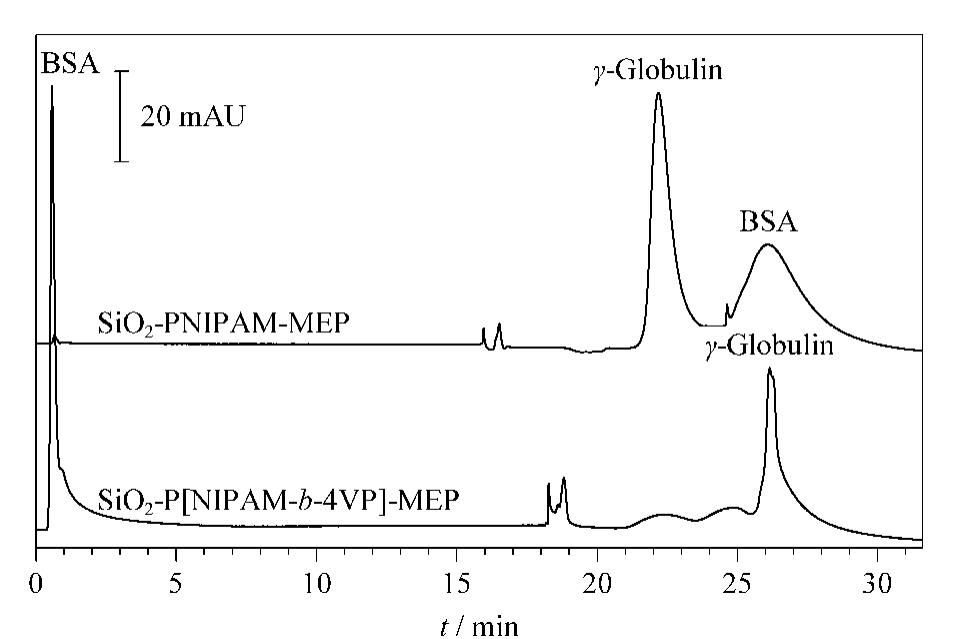
温敏亲和色谱对BSA和*γ*-球蛋白的分离色谱图

从图4可以看出,BSA和*γ*-球蛋白均可在线性温敏亲和色谱固定相SiO_2_-PNIPAM-MEP上保留,且BSA的保留较*γ*-球蛋白强。虽然该固定相对二者可实现基线分离,但对*γ*-球蛋白的特异选择性较差。从嵌段温敏亲和色谱固定相SiO_2_-P[NIPAM-*b*-4VP]-MEP对二者的色谱分离图可以看出,BSA在该固定相上不保留,仅洗脱得到*γ*-球蛋白一个色谱峰,表明嵌段温敏亲和色谱固定相SiO_2_-P[NIPAM-*b*-4VP]-MEP对*γ*-球蛋白表现出特异的选择性,一步纯化其质量回收率为92.7%。测定了*γ*-球蛋白在SiO_2_-PNIPAM-MEP和SiO_2_-P[NIPAM-*b*-4VP]-MEP两种固定相的平均最大吸附量分别为(43.1±1.3) mg/g和(71.5±2.1) mg/g(*n*=3),后者较前者对*γ*-球蛋白的吸附量提高了近1倍。这是由于采用温敏嵌段共聚物P[NIPAM-*b*-4VP]为间隔臂时,P4VP是一种疏水性的线性聚合物链,不仅可以调节温敏嵌段共聚物的疏水性,而且在温度高于PNIPAM的LCST时,PNIPAM发生脱水卷曲收缩,由于P4VP嵌段的存在,使聚合物末端构建的亲和配体MEP更容易暴露在外而不被包埋在聚合物内部,有助于配基与*γ*-球蛋白的结合。此外,P4VP主链上有大量的吡啶基,可与*γ*-球蛋白产生疏水作用力,故能够提供更多的吸附位点^[17,21]^,因此嵌段共聚温敏亲和色谱固定相对*γ*-球蛋白具有更高的吸附量。

### 2.3 温度的影响

如前所述,温度可影响温敏色谱固定相表面的亲疏水性,从而影响蛋白质在温敏色谱固定相上的保留与洗脱。本文详细研究了不同温度下嵌段温敏亲和色谱固定相对*γ*-球蛋白和BSA色谱分离的影响,结果如图5所示。从图5可以看出,采用50 mmol/L Tris-HCl+0.6 mol/L NaCl(pH 8.0)为流动相B液,当洗脱温度为40 ℃时,仅有79.8%的*γ*-球蛋白被洗脱。随着温度的降低,更多的*γ*-球蛋白被洗脱,色谱峰变得越来越高。当温度降低至5 ℃时,*γ*-球蛋白的色谱峰变得更尖锐。这表明5 ℃时,温敏色谱固定相的表面亲水性显著增强,有利于蛋白质的洗脱,同时,低温亦使*γ*-球蛋白与亲和配基MEP的结合常数减小,更有利于*γ*-球蛋白的洗脱^[19]^。此外,还测定了该固定相在10、20、30、40 ℃时对*γ*-球蛋白的最大吸附量,分别为(6.1±1.4)、(21.1±0.8)、(46.6±1.2)和(71.5±2.1) mg/g(*n*=3),表明该嵌段温敏亲和色谱固定相对*γ*-球蛋白的吸附量随温度的升高而增大,进一步证明高温更有利于*γ*-球蛋白吸附,而低温更有助于*γ*-球蛋白的洗脱。

**图5:**
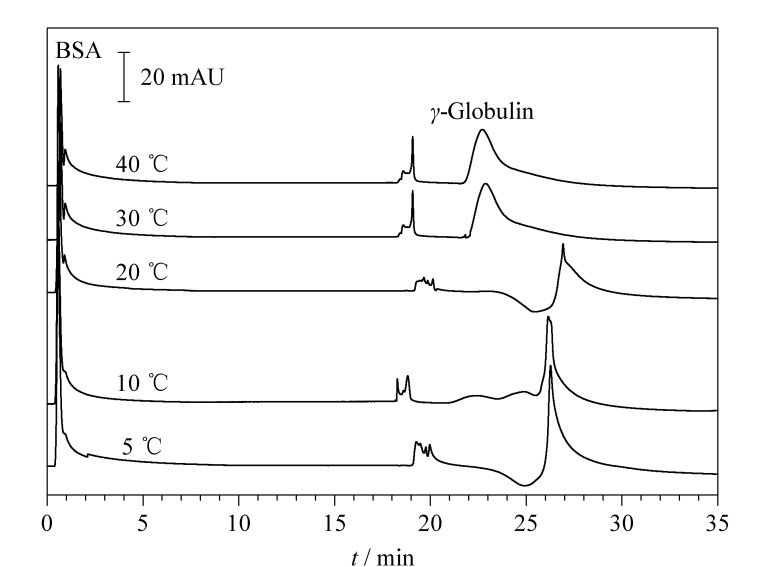
洗脱温度对*γ*-球蛋白和BSA色谱分离的影响

### 2.4 盐浓度的影响

亲和色谱中,在流动相中添加一定浓度的盐有利于蛋白质的洗脱。我们在流动相B液中添加不同浓度的NaCl,研究了盐浓度对*γ*-球蛋白洗脱的影响,结果如图6所示。当流动相B液中不含NaCl时,无法将*γ*-球蛋白从温敏亲和色谱固定相上洗脱下来。当流动相中添加0.2 mol/L NaCl时,仅有少量*γ*-球蛋白被洗脱下来。随着NaCl浓度增大,*γ*-球蛋白的色谱峰逐渐增高,表明越来越多的*γ*-球蛋白被洗脱。当NaCl的浓度为0.6 mol/L时,*γ*-球蛋白的回收率达到了92.7%±1.2%(*n*=3); NaCl浓度为1.0 mol/L时,其回收率大于95%。考虑到高盐浓度对抗体蛋白稳定性^[22]^及后处理的影响,采用温敏亲和色谱分离BSA和*γ*-球蛋白时,选择在流动相B液中添加0.6 mol/L的NaCl。

**图6:**
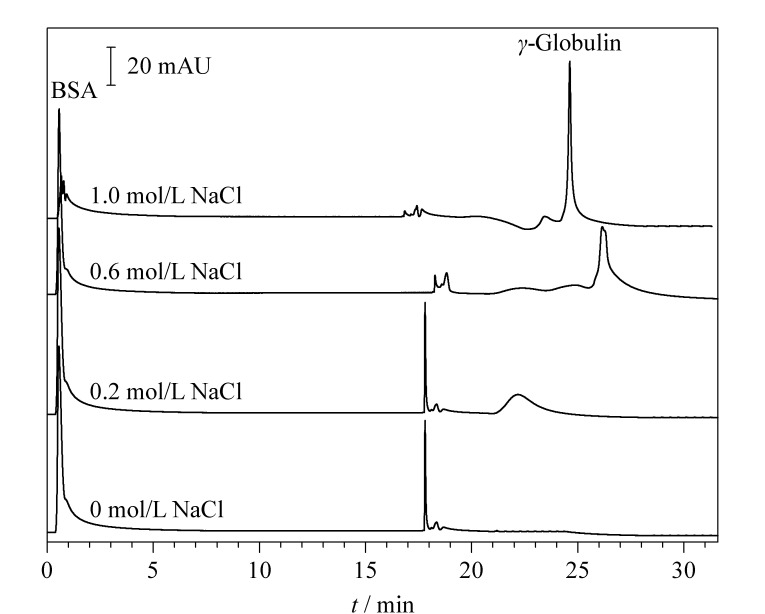
流动相中NaCl浓度对*γ*-球蛋白洗脱的影响

### 2.5 应用

将嵌段共聚温敏亲和色谱固定相SiO_2_-P[NIPAM-*b*-4VP]-MEP用于人血清中免疫球蛋白lgG的分离纯化,在40 ℃进样,5 ℃时进行洗脱,分离结果和电泳分析如图7所示。由图7a可以看出,杂蛋白无法在该色谱柱中保留,仅有IgG保留,而且仅通过调节温度和洗脱液中NaCl浓度就可以从血清中纯化IgG,如图7b所示,平行3次纯化的IgG的平均纯度为97.4%±0.7%(*n*=3)。

**图7:**
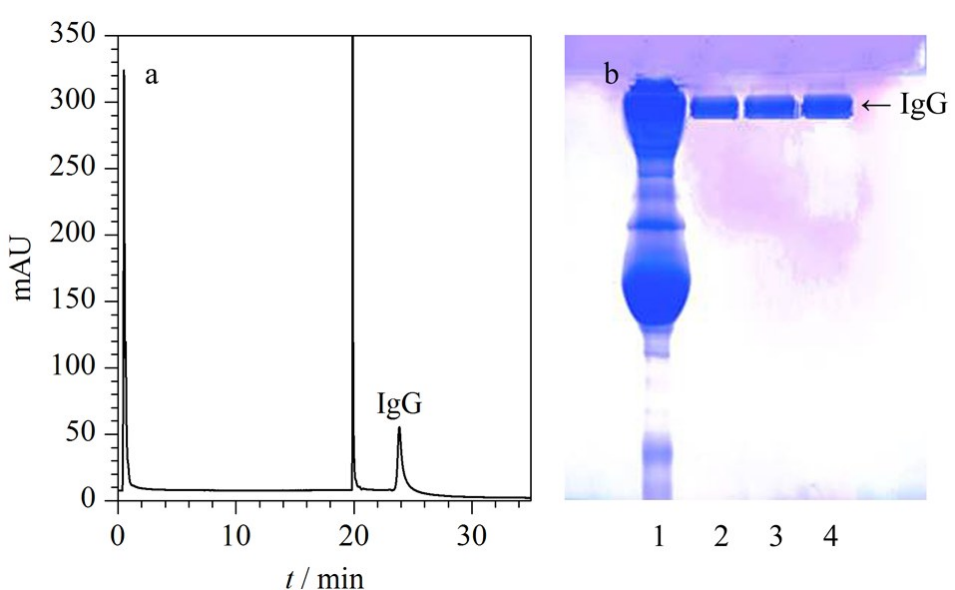
温敏亲和色谱固定相SiO_2_-P[NIPAM-*b*-4VP]-MEP 对人血清中IgG的(a)色谱分离图及(b)SDS-PAGE电泳分析结果

## 3 结论

本研究将NIPAM与4-VP共聚以调节温敏嵌段共聚物P[NIPAM-*b*-4VP]的亲疏水性,所制备的嵌段共聚温敏亲和色谱固定相SiO_2_-P[NIPAM-*b*-4VP]-MEP不仅对抗体具有特异的选择性,而且可提供更多的作用位点,使抗体的吸附容量提高了1倍。所构建的嵌段共聚温敏亲和色谱仅通过调节温度和流动相盐浓度即可实现对抗体一步分离纯化,操作简单,绿色环保,从根本上解决了传统色谱采用酸性洗脱易使抗体蛋白变性失活的难题。该技术在抗体药物的分离纯化和工业生产中具有重要的应用价值。
